# Stem cell depletion by inflammation-associated miR-155

**DOI:** 10.18632/aging.101374

**Published:** 2018-01-23

**Authors:** Takeshi Teramura, Yuta Onodera

**Affiliations:** 1 Division of Cell Biology for Regenerative Medicine, Institute of Advanced Clinical Medicine, Kindai University, Osaka, Japan

**Keywords:** stem cell aging, inflammation, ROS, miRNA

MicroRNAs (miRNAs), non-coding RNAs of 19–25 nucleotides, play critical roles in various cellular processes such as proliferation, differentiation, and cell viability. During inflammation, the expression of certain miRNAs is upregulated, and these miRNAs contribute to some inflammation-induced degenerative reactions. Among the inflammation-associated miRNAs identified, miR-155 is highly conserved one across vertebrate species and is thought to be one of the important miRNA involved in the inflammatory response. Pro-inflammatory signals, such as interleukin (IL)-1, IL-6, and tumor necrosis factor (TNF)-α upregulate the expression of miR-155. Recently, it has been discovered that expression of miR-155 is increased in some chronic CNS disorders [1]. Furthermore, inhibiting miR-155 activity with complementary anti-miRNA oligonucleotides reduces impairment in animal models of CNS disorders. Moreover, in aged individuals [2] and tissues [3], upregulation of miR-155 expression is demonstrated. Since chronic inflammation is often associated with normal and pathological aging [4], the idea that miR-155 expression is activated in aged tissues and involves in tissue degeneration is reasonable.

In the inflammatory condition, stem cells are excessively activated or/and accumulate cytotoxic molecules such as reactive oxygen species (ROS). These actions could lead to depletion of the stem cell pool and, as a result, induce tissue degeneration [5]. In the recent studies, it has been reported that miR-155 is responsible for both induction of differentiation [1] and generation of ROS in the somatic stem cells [3]. In the neural stem cells (NSCs), overexpression of miR-155 resulted in disruption of stem cell self-renewal-associated genes *Musashi1*, *Hes1*, and *Bmi1*. The miR-155 expressing NSCs failed to form neurospheres, and their proliferation was reduced. On the contrary, inhibition of miR-155 suppressed IL-1-induced differentiation in the NSCs. Importantly, it has been shown that these reactions were conserved in human NSCs derived from induced pluripotent stem (iPS) cells [1].

**Figure 1:**
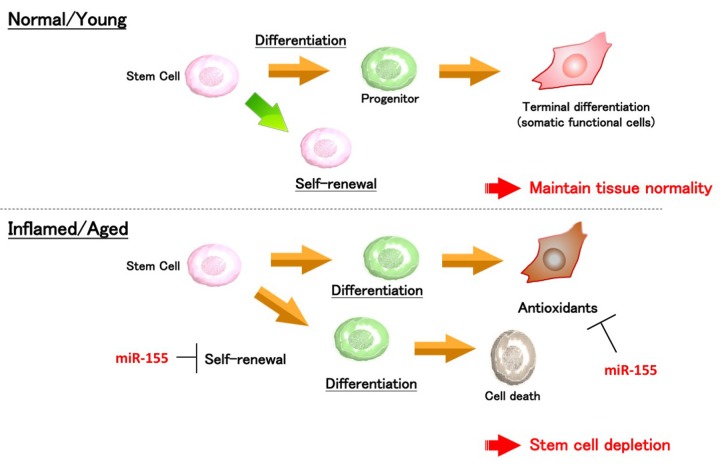
Schematic representation of the miR-155-mediated stem cell deterioration. In the inflammatory condition, upregulated miR-155 activates stem cell differentiation, but expression of antioxidant-related genes is suppressed by miR-155. Thus, it is thought that excessive inflammation and miR-155 expression could be a risk for stem cell depletion and disturbance of tissue integrity.

On the other hand, accumulation of ROS is a well-known feature of aging and is the direct cause of stem cell degeneration. Since ROS can form a positive feedback loop with inflammatory cytokines, it is essential to find the critical mediator connecting inflammation and ROS generation in order to develop a way to manage it. In the recent paper, it has been demonstrated that miR-155 suppressed the anti-oxidant genes *Nfe2l2*, *Sod1*, and *Hmox1* and triggered ROS accumulation in the mesenchymal stem cells (MSCs). When miR-155 expression is suppressed, IL-1β induced ROS generation was moderated. Consistent with these notions, deletion of miR-155 genes by the CRISPR/Cas9 system brought about reduction of ROS levels in the MSCs *in vivo* [3]. Based on these results, an important hypothesis was proposed: miR-155 induce ROS generation by suppressing antioxidant gene expressions, which is the phenomenon observed in inflamed and/or aged tissues. The aforementioned two studies with NSCs and MSCs suggest that miR-155 is a responsible molecule for inflammation-associated stem cell dysfunction. A central question in these studies was how miR-155, just a single miRNA, regulated multiple genes involving different biological events. We hypothesized that miR-155 affects expression of these genes through targeting of a common master transcription factor(s). Analysis using public digital databases identified that a CCAAT/enhancer-binding protein, C/EBPβ, is one of the molecules mediating the miR-155-induced stem cell dysfunctions. Inhibition of C/EBPβ resulted in induction of differentiation in NSCs and ROS generation in MSCs. Furthermore, the expression level of C/EBPβ was strongly attenuated by miR-155. These observations provided the scheme of process connecting aging-associated inflammation and stem cell deteriorations: 1) inflammation activates miR-155 expression, 2) miR-155 blockades C/EBPβ expression, and 3) downregulation of C/EBPβ results in decreased expression of its downstream genes needed for antioxidation and stem cell self-renewal (Figure 1).

Although we presumed that miR-155 is an inflammation-associated mediator in the above two studies, miR-155 expression could also be upregulated by hypoxic conditions or loss of its transcriptional repressors. Now, it has been shown that aging is a process accompanied by a general decrease in O_2_ supply in tissues [6]. Furthermore, it was also reported that expression of Notch1, which is a major repressor of miR-155 transcription, was significantly downregulated in aged mice [7]. These evidences show that aged tissue is a very supportive environment for miR-155 expression, and therefore miR-155 could be an important player for aging/inflammation-related stem cell deterioration. Now, miRNA therapy modulating pathogenic miRNA expression by antisense inhibition has become a promising option for the management of various diseases. Therapies using miR-155 inhibitors may provide novel strategies for managing various inflammation-associated diseases, aging, and their related stem cell depletion.
